# Metabolic Crosstalk between Liver and Brain: From Diseases to Mechanisms

**DOI:** 10.3390/ijms25147621

**Published:** 2024-07-11

**Authors:** Xiaoyue Yang, Kangli Qiu, Yaoyao Jiang, Yumei Huang, Yajuan Zhang, Yunfei Liao

**Affiliations:** Department of Endocrinology, Union Hospital, Tongji Medical College, Huazhong University of Science and Technology, Wuhan 430022, China

**Keywords:** liver, brain, interorgan crosstalk, metabolism, hepatokines, metabolites, autonomic nervous system

## Abstract

Multiple organs and tissues coordinate to respond to dietary and environmental challenges. It is interorgan crosstalk that contributes to systemic metabolic homeostasis. The liver and brain, as key metabolic organs, have their unique dialogue to transmit metabolic messages. The interconnected pathogenesis of liver and brain is implicated in numerous metabolic and neurodegenerative disorders. Recent insights have positioned the liver not only as a central metabolic hub but also as an endocrine organ, capable of secreting hepatokines that transmit metabolic signals throughout the body via the bloodstream. Metabolites from the liver or gut microbiota also facilitate a complex dialogue between liver and brain. In parallel to humoral factors, the neural pathways, particularly the hypothalamic nuclei and autonomic nervous system, are pivotal in modulating the bilateral metabolic interplay between the cerebral and hepatic compartments. The term “liver–brain axis” vividly portrays this interaction. At the end of this review, we summarize cutting-edge technical advancements that have enabled the observation and manipulation of these signals, including genetic engineering, molecular tracing, and delivery technologies. These innovations are paving the way for a deeper understanding of the liver–brain axis and its role in metabolic homeostasis.

## 1. Introduction

In the past thirty years, the global incidence of type 2 diabetes [1] and non-alcoholic fat disease (NAFLD) [2] has risen rapidly. These pervasive metabolic diseases are characterized by overlapping features, including insulin resistance and fatty accumulation in the liver [1]. Historically, research has concentrated on the causes of peripheral metabolic dysfunctions, with scant attention paid to the role of crosstalk between peripheral organs and the central nervous system (CNS) in preserving systemic metabolic homeostasis. Interorgan crosstalk is a key way to mobilize organs against environmental and physiological changes to ensure overall energy homeostasis. The liver’s role in clearing detrimental cerebral proteins during senescence has suggested the contribution of the liver to central metabolic regulation [3]. Both the brain, especially the hypothalamus, and liver are critical metabolic organs responsible for discovering, relaying and reacting to signals that emanate from the systemic energy metabolism. In fact, disturbances of hepatic and cerebral metabolism are common in numerous metabolic illnesses [4,5]. The liver not only functions as a supplier of essential nutrients to the brain but also serves as a crucial organ for detoxifying splanchnic blood. It was not recognized until the last decade that the liver and brain also engage in a distinct metabolic dialogue, leading to the coinage of the new term “liver–brain axis” [6,7].

The metabolic dialogue along the liver–brain axis is bidirectional, with both afferent (“liver-to-brain” communication) and efferent (“brain-to-liver” communication) directions. Hepatokines, metabolites and afferent sensory nerves transmit metabolic stimuli from the liver to the brain, while neural signals from the CNS, after integrating peripheral cues, influence the macronutrients metabolism of the liver.

This review discusses the current understanding of metabolic signaling across the liver–brain axis. First, we analyze liver–brain comorbidity, whose clinical manifestations and pathogenesis are indicative of the close connection between the liver and the brain. Hepatokines and metabolites that deliver hormonal information from the liver to the CNS are described in Section 2 and Section 3. Discussed in Section 4 is the neurological connection between the hypothalamus and the liver. Lastly, we present an overview of innovative strategies for mapping metabolic crosstalk between the liver and brain, including tracing methods and transmitting techniques.

## 2. Interconnected Diseases of the Liver and Brain

**Figure 1:**
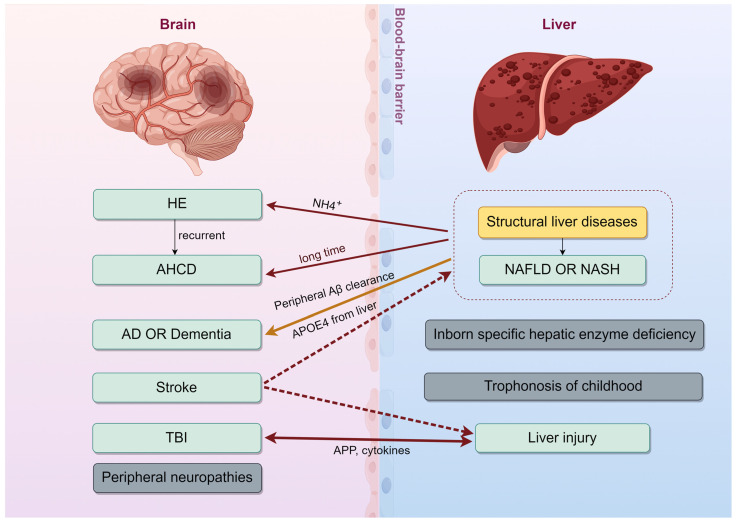
The brain and liver are related to each other in the pathogenesis of many diseases. HE, hepatic encephalopathy; AHCD, acquired hepatocerebral degeneration; AD, Alzheimer’s disease; TBI, traumatic brain injury; NAFLD, non-alcoholic fatty liver disease; NASH, non-alcoholic steatohepatitis; Aβ, amyloid-β; APOE4, apolipoprotein E4. This figure was created using Figdraw.

The interplay between liver and brain has been recognized for centuries. Hepatic encephalopathy (HE) is perhaps the most well-known manifestation of this connection, characterized by neuropsychiatric symptoms stemming from liver dysfunction. Beyond HE, however, the intricate pathological links between liver and brain seem obscure. This part focuses on the interconnected diseases of the liver and brain, providing an overview of the clinical relationship between liver and brain (Figure 1).

### 2.1. Neurological Syndrome Linked to a Liver Disease

As a concept, metabolic diseases of the nervous system means the manifestations of systemic metabolic diseases in the nervous system [8]. Neurological syndromes associated with a liver disease encompass a spectrum of conditions, including CNS function related to structural liver diseases, neurological consequences caused by inborn specific hepatic enzyme deficiency, trophonosis during childhood that affect the liver and CNS, and peripheral neuropathies associated with liver conditions. Given the prevalence of these disorders, neurologic manifestations other than structural liver diseases (e.g., NFALD, liver fibrosis, liver cirrhosis, hepatocellular carcinoma et al.) cannot be addressed due to limited space.

#### 2.1.1. Hepatic Encephalopathy (HE)

HE stands as a quintessential example of how liver failure can precipitate alterations in brain function [8]. Impaired liver function results in inadequate detoxification, thus allowing the entry of neurotoxins, such as ammonia, manganese, and other harmful substances, into the cerebral circulation. As of now, elevated serum ammonia levels have been central to our comprehension of HE, and therapy strategies remain aimed at lowering ammonia concentrations in the body [5].

#### 2.1.2. Acquired Hepatocerebral Degeneration (AHCD)

AHCD arises from recurrent hepatic encephalopathy, or multiple metabolic disorders over a long period of time [9]. The onset of AHCD is generally insidious, with the main manifestations of mental disorders, cognitive decline, and Parkinson’s disease-like syndromes, which are easily misdiagnosed as neurodegenerative diseases [10]. AHCD differs from hepatic encephalopathy with a solid lesion in the brain [11,12]. Pathologically, it is marked by neuronal loss and the accumulation of glycogen granules in the cytoplasm of the basal ganglia, along with abnormal hypoperfusion in some specific brain regions. Studies have shown that AHCD is associated with a variety of metabolic abnormalities, such as ammonia, aromatic amino acids, manganese, etc. The neurotoxic effects of manganese are thought to be causative in AHCD [13], with impaired hepatobiliary clearance leading to higher serum and cerebrospinal fluid manganese levels in patients with AHCD [14]. Manganese deposition in the brain often causes diffuse brain parenchymal degeneration [12].

#### 2.1.3. Stroke

Patients with liver disease are prone to both bleeding and thrombophilia due to the impaired synthesis of coagulation and anticoagulant factors [15], thus increasing their risk of stroke. Whether NAFLD increases the risk of stroke is currently under intense investigation. Research with 79,905 participants (including 24,874 NAFLD participants) indicated that those with NAFLD had a 16% higher risk of ischemic stroke than those without NAFLD at baseline [15]. A mendelian randomization study suggests that the potential causal effect of NAFLD on ischemic stroke may be specific to the small vessel occlusion subtypes and large artery atherosclerosis [16]. As shown above, the liver–brain axis plays a vital role in stroke and NAFLD. However, contrasting findings from a European cohort study of 120,795 adults with diagnosed NAFLD or NASH, adjusting for established cardiovascular risk factors, revealed no association between NAFLD/NASH diagnosis and stroke risk [17]. Similarly, a small observational study, which included 1601 patients, also showed that the presence of NAFLD did not exert an impact on post-stroke disability or mortality outcomes [18].

### 2.2. Neurodegenerative Diseases and the Liver

#### 2.2.1. Liver’s Role in Neurodegeneration

An emerging therapeutic concept for neurodegenerative diseases, brain energy rescue [3] highlights similarities in the pathogenesis between neurodegenerative diseases and metabolic syndrome. More importantly, the liver may be a key regulator of both. Metabolomics have shown that the liver was the earliest affected organ during the amyloid pathological cascade in APP/PS1 mice at 5 months of age [19], manifesting as hepatic hypometabolism and perturbed metabolites mainly involved in amino acid metabolism, nucleic acid metabolism, fatty acid metabolism, energy metabolism, and ketone body metabolism. In human populations, liver dysfunction also correlates with cognitive decline and AD [20]. The clinical and pathophysiological association of NAFLD and fibrosis with incident dementia and cognition has been widely documented [21,22,23]. Low-density lipoprotein receptor-related protein 1 (LRP-1) and amyloid-β (Aβ) levels in the liver tissue of rats [24] and mice [25] with NAFLD were reduced; these decreases correlated inversely with Aβ levels in the brain and plasma as well as cognitive function. Reduced peripheral LRP-1 causes brain Aβ accumulation and cognitive impairment in NAFLD by mediating the negative effects of NAFLD on peripheral Aβ clearance [24]. Furthermore, clinical observation data demonstrated that cirrhosis patients exhibited increased plasma levels of Aβ40 and Aβ42 compared to controls with normal liver function [26,27].

#### 2.2.2. Liver’s Clearance of Aβ

The aggregation of cerebral Aβ accumulation, consequent to impaired Aβ clearance, is a central event in the pathogenesis of AD. Strategies for AD treatment have primarily concentrated on clearing Aβ from the CNS, but these attempts have not yielded significant therapeutic benefits [28]. The physiological capacity of peripheral organs to clear brain-derived Aβ is pivotal in attenuating brain Aβ burden [29]. A significant proportion of brain-derived Aβ, estimated to be 40–60%, is transported to the periphery through the blood–brain barrier (BBB), lymphatic pathways, etc. [30]. The liver, as a major organ tasked with the clearance of metabolites in the periphery, eliminates a considerable portion of Aβ42 (13.9%) and Aβ40 (8.9%) from the bloodstream [26]. This clearance was reduced by the down-regulation of the hepatocyte Aβ receptor LRP-1 with increasing age.

#### 2.2.3. Liver-Derived APOE in AD Pathogenesis

There is an abundant expression of apolipoprotein E (APOE), a hallmark molecule for AD, in both the brain and the liver. ApoE mRNA expression in the brain is 1/3 of that in the liver, and astrocytes are the main synthesis sites [31]. The ε2, ε3 and ε4 alleles of the *APOE* gene make up the proteins of three ApoE isoforms, ApoE2, ApoE3, and ApoE4, in which E3 is a protective factor and E4 is a risk factor for Alzheimer’s disease [32].

It has been demonstrated in mouse models that liver-expressed apoE4, independent of brain-expressed apoE4, has a separate impact on synaptic plasticity and cognition by impairing cerebrovascular function [33]. To be specific, Liu et al. [33] and Lam et al. [34] created conditional mouse models where human APOE3 or APOE4 were expressed in the liver but not in the brain. When these mice were bred with APP/PS1 model mice, they observed that the presence of apoE4 in the liver worsened brain Aβ deposition and led to cerebrovascular dysfunction, while apoE3 had the opposite effect, reducing Aβ deposition.

Some hepatic indicators can reflect the progression of amyloid pathology in the brain; however, routine liver function tests fail to detect the liver’s Aβ clearance capabilities. Liver LRP-1, Aβ and APOE may be used for the early diagnosis of AD. Peripheral Aβ clearance and apoE4 blockade that are targeted at the liver provide a strong rationale to treat AD.

### 2.3. Hepatic Responses to Cerebral Lesions

Brain alterations caused by liver diseases are frequently observed. On the contrary, is it feasible that a brain injury can affect liver metabolism? This issue is critical in liver transplants from brain-dead donors, as well as the prognosis for brain damage.

Traumatic brain injury (TBI) is a serious public health issue, with a mortality rate of 20% to 30% [35] and affecting 27 to 60 million individuals annually [36]. The great frequency of drug-induced liver damage during hospitalization for brain injury has been clinically documented [37]. The liver contains the highest macrophage density among organs and synthesizes most chemokines and cytokines in serum following brain trauma. In the aftermath of acute brain damage, chemokine production in liver triggers neutrophil recruitment and subsequent hepatic damage [37]. The liver itself manifests an increase in enzyme markers of liver tissue injury and acute-phase proteins (APP). On the one hand, chemokines from the liver amplify the inflammatory response from the CNS to the whole body. On the other hand, hepatic inflammation alleviates CNS damage by promoting the migration of circulating immune cells into the injured brain. These molecular signals from the liver, in response to brain injury, are instrumental in the peripheral regulation of brain function.

Recently, attention has been paid to a protein whose expression is diminished in the liver after brain injury. Employing established mouse models of TBI, Zhu et al. observed a prompt decrease in hepatic soluble epoxide hydrolase (sEH) levels after TBI, and it subsequently returned to baseline. The serum level of 14,15-EET (epoxyeicosatrienoic acid) is inversely related to hepatic sEH activity. 14,15-EET, rapidly crossing the blood–brain barrier, mimics the neuroprotective effect of hepatic sEH deficiency by facilitating the emergence of A2 phenotype astrocytes in response to TBI [38].

These findings underscore the neuroprotective capacity of the liver in cerebral lesions. Most of the TBI treatment was grounded in neuroprotective measurements to curtail inflammation and secondary brain damage [39]. Given that focal brain lesions elicit a fast hepatic reaction [40], it is imperative to consider liver injury after brain lesions and avert secondary brain injury from the perspective of hepatic molecular signaling.

## 3. Hepatokines Which Act on the Brain

It has recently been discovered that the liver produces a variety of humoral substances as endocrine moderators [41,42]. The role of several hepatokines in the pathology of obesity, diabetes, and NAFLD has been studied [41,43]. Some of these hepatokines have been found to affect other diseases, especially encephalopathic ones such as AD and TBI. Moreover, some of these circulating factors transmit metabolic messages from the liver to the brain, thereby modulating body weight and food intake [44,45]. Receptors of a few hepatokines have been identified in the CNS [46,47]. While the precise neural targets and receptors for numerous hepatokines remain to be fully elucidated, emerging evidence points to their participation in central metabolic regulatory processes [48,49]. Here we summarize the current understanding of hepatokines that exert effects on the brain, outline their known receptors, and discuss their action on the brain (Table 1).

### 3.1. FGF21

**Figure 2:**
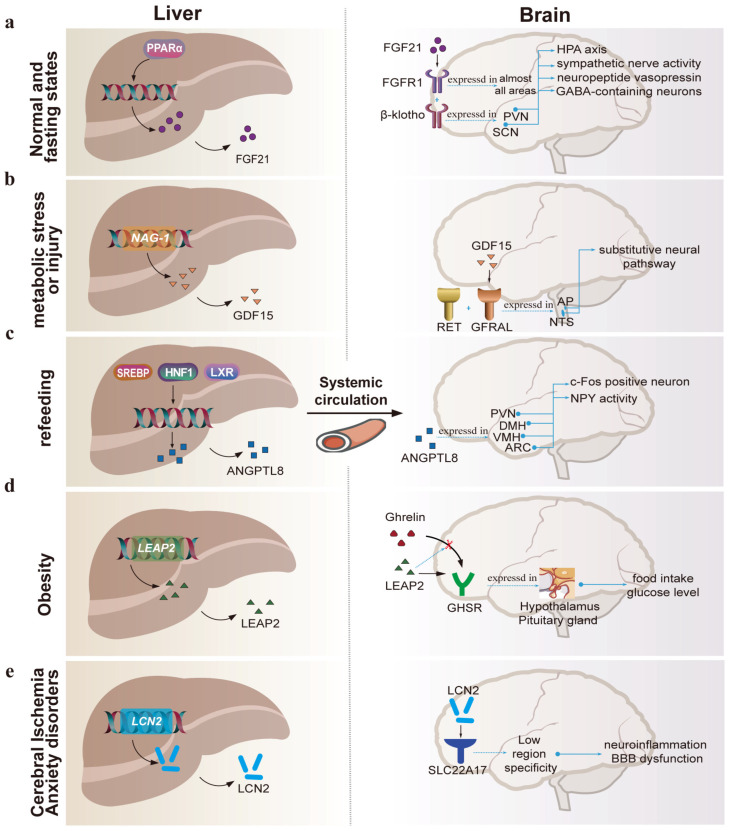
Hepatokine signaling from liver to brain. (**a**) FGF21, as a hepatokine, acts on the hypothalamus to regulate the HPA axis, GABA-containing neurons, and sympathetic nerve activity. (**b**) GDF15 is mainly secreted by the liver under injury or metabolic stress as an endocrine signal that initiates emergency neural circuits. (**c**) Both peripheral and central ANGPTL8 administration reduces c-Fos positive neuronal expression in the DMH and alters NPY activity to reduce food intake. ANGPTL8 is widely expressed in the PVN, DMH, VMH, and ARC. (**d**) LEAP2 serves as a liver-derived antagonist of the ghrelin receptor, and its secretion is suppressed by fasting. (**e**) Stress triggers the release of LCN2 from the liver, which in turn contributes to the development of anxiety-like behavior in mice. FGF21, fibroblast growth factor 21; PVN, paraventricular nucleus; SCN, suprachiasmatic nucleus; HPA, hypothalamic–pituitary–adrenal; AP, area postrema; NTS, nucleus of the solitary tract; RET, rearranged during transfection; GFRAL, glial-derived neurotropic factor receptor-alike; DMH, dorsal medial nucleus; VMH, ventral medial nucleus; ARC, arcuate nucleus; GHSR, growth hormone secretagogue receptor; SLC22A17, solute carrier family 22 member 17; BBB, blood–brain barrier; GDF15, growth differentiation factor; ANGPTL8, angiopoietin-like proteins; LEAP2, liver-enriched antimicrobial peptide-2; LCN2, lipocalin-2.

The liver is thought to be the primary origin of circulating fibroblast growth factor 21 (FGF21) [50], a hormone induced by peroxisome proliferator-activated receptor alpha (PPARα) in the liver [51,52]. A receptor complex made up of a classic FGF receptor (FGFR1) and the essential FGF co-receptor (β-klotho) interprets how FGF21 communicates with cells [53]. FGF21 in the blood can penetrate the blood–brain barrier [53] and can be detected in human cerebrospinal fluid [54]. FGFR1 is spread throughout the CNS [46], but the co-receptor β-klotho is predominantly expressed in a few areas that control energy homeostasis [46,55], including the suprachiasmatic nucleus (SCN) [56] and the paraventricular nucleus (PVN) [57] (Figure 2a).

FGF21 may have the potential to treat diabetes and obesity by acting on the CNS. Central FGF21 treatment increased metabolic rate and hepatic insulin sensitivity. These metabolic processes are accompanied by the enhancement of sympathetic nerve activity [53,58], the alteration of circadian behavior, and the increase in glucocorticoid concentrations [56]. FGF21 has been reported to regulate metabolism centrally in the following ways: Firstly, hepatic FGF21 affects peripheral metabolism, probably mediated by inducing the activation of the hypothalamic–pituitary–adrenal (HPA) axis [57] and suppressing the expression of the neuropeptide vasopressin in the SCN [59]. Secondly, it stimulates sympathetic nerve activity via a process involving the neuropeptide corticotropin-releasing factor [58]. Thirdly, FGF21 also stimulates GABA-containing neurons in the lateral hypothalamic region and zona incerta to protect against obesity [60]. Fourthly, FGF21, as a humoral regulator of sugar and alcohol appetite, activates glutamatergic neurons in the VMH to lower sucrose intake [61,62]. Additionally, through an amygdalo-striatal circuit [63], it decreases alcohol consumption and raises water consumption [64]. Taken together, these findings provide a unified description for how FGF21 works at a central site (Figure 2a).

**Table 1:** Hepatokines which act on the brain. Molecular weights are calculated from https://www.uniprot.org (accessed on 26 May 2024).

Hepatokines	Molecular Weight	Concentration in Human Blood	Receptors	Central Site of Receptor Expression	Effects on the Brain	Diseases with Therapeutic Potential
Apolipoprotein E (APOE)	34 kDa	0.03~0.05 g/L [65]	Low density lipoprotein receptor(LDLR) family; LDL receptor-related protein 1(LRP1)	Low region specificity	Maintains cholesterol homeostasis of brain; liver-expressed apoE4 exacerbated brain Aβ deposition and cerebrovascular dysfunction, whereas apoE3 reduced it.	Alzheimer’s disease [33,34]
Fibroblast growth factor 21 (FGF21)	19.5 kDa	200~300 pg/mL [66]	FGF receptor (FGFR1); FGF co-receptor (β-klotho)	FGFR1 is spread throughout the nervous system, but co-receptor β-klotho is predominantly expressed in hypothalamus, hippocampal region, subiculum, and amygdala [55]	Regulates energy homeostasis, via activation of the hypothalamus–pituitary–adrenal axis.	Obesity; NAFLD; Diabetes mellitus [57]
Growth differentiation factor 15 (GDF15)	24.8 kDa	100~1200 pg/mL [47]	Glial-derived neurotropic factor receptor-a like (GFRAL); co-receptor rearranged during transfection (RET)	The area postrema (AP); the nucleus of solitary tract (NTS) [67]	Conveys peripheral metabolic messages to the brain where it activates substitutive neuronal pathways to adapt to shifting energy demands; reduces food intake and body mass.	Diabetes mellitus; Obesity; NAFLD [68,69]
Tsukushi (TSK)	34 kDa	18–49 ng/mL [70]	Not clear yet	Not clear yet	Functions as a liver-derived feedback hormone that attenuates energy expenditure by engaging in crosstalk with the CNS in hypermetabolic states.	Metabolic disease [49]
Angiopoietin-like protein 8 (ANGPTL8)	22.5 kDa	~300 pg/mL [71]	Leukocyte immunoglobulin-like receptor B3 (LILRB3) [72]	Low region specificity and vasculature (mainly)	Is involved in the regulation of appetite.	Anorexia; Diabetes mellitus; Obesity; NAFLD [73]
Insulin-like growth factor 1 (IGF-1)	7.6 kDa	82~487 ng/mL [74]	Insulin like growth factor 1 receptor (IGF1R)	Low region specificity	Mediates brain growth and development; functions as an anti-apoptotic agent by enhancing cell survival.	Disorders related to brain development [75]; Traumatic brain injury [76]; Age-Related Neurological Conditions [77,78]
Energy Homeostasis Associated gene (ENHO) (Adropin)	5.0 kDa	3.4~4.5 ng/mL [79]	Not clear yet [80]	Not clear yet	Regulates endothelial cells and maintains blood–brain barrier integrity.	Transient Ischemic Stroke [81,82]; Aging-related neuropathology [83]
Liver-enriched antimicrobial peptide-2 (LEAP2)	23 kDa	5~20 ng/mL [84]	Growth hormone secretagogue receptor (GHSR)	Hypothalamus, Pituitary gland	Endogenous antagonist of Ghrelin Receptor, thus preventing the effects of ghrelin; regulator of food intake, glucose level and body weight.	Obesity [85]
Lipocalin-2 (LCN2)	22.6 kDa	590 µg/L ^1^	Solute carrier family 22 member 17 (SLC22A17)	Low region specificity	Induces neuroinflammation and blood–brain barrier dysfunction [86]; induces anxity-like behavior through Lcn2 receptors in the medial prefrontal cortex (mPFC).	Cerebral Ischemia [87]; Anxiety disorders [88]; Neurodegenerative diseases [89]

^1^ These data are from PeptideAtlas (https://peptideatlas.org/, accessed on 26 May 2024).

### 3.2. GDF15

Under normal metabolic conditions, growth differentiation factor 15 (GDF15) can be found in almost all tissues, and is not substantially expressed in the liver [44,90]. However, in response to a high-fat diet or obesity, the liver emerges as the primary source of plasma GDF15 [91]. The intraventricular injection of GDF15 reduced food consumption and caused weight loss by 10–24% in the model organism after 5–6 weeks [91], indicating that the brain may serve as a site of action for GDF15 to modulate feeding. GDF15-induced weight loss surpasses that achieved through caloric restriction alone [92,93]. GDF15 can reverse the compensatory decrease in energy expenditure, making it a promising candidate for avoiding weight regain after weight loss. Four teams simultaneously and independently localized glial-derived neurotropic factor receptor-alike (GFRAL), the receptor for GDF15, to two regions within the hindbrain in mice [47,94,95,96] in 2017. GFRAL mRNA was detected [97] in specific brain areas [67] (Figure 2b), namely the area postrema (AP) and the nucleus of the solitary tract (NTS). GFRAL, together with the co-receptor rearranged during transfection (RET) [94,98], triggers intracellular signaling to specifically activate GFRAL-expressing neurons in AP and NTS, which in turn influence neurons within the parabrachial nucleus and central amygdala [94].

Interestingly, GDF15 appears to be an emergency hormonal signal from the liver [99], conveying peripheral metabolic messages to the brain, where it activates substitutive neuronal pathways [69] to adapt to shifting energy demands under metabolic stress (Figure 2b). Furthermore, this adaptive response is unrelated to other appetite-regulating hormones (e.g., leptin, growth hormone-releasing peptide, and glucagon-like peptide 1) to a large extent [68]. In an animal model of lipopolysaccharide (LPS)-induced inflammatory injury, the GDF15 blockade resulted in lower norepinephrine efflux from the output ganglia and reduced hepatic and plasma triglyceride levels [100]. Thus, GDF15 may enhance organic tolerance during metabolic imbalances as a liver–brain axis mediator.

### 3.3. ANGPTL8

Angiopoietin-like proteins (ANGPTLs) are a group of secretory glycoproteins that structurally resemble angiopoietins. They are recognized as the key regulators of lipid metabolism, since they affect the activity of lipoprotein lipase (LPL) through post-translational modifications, thereby increasing circulating triglyceride levels [101,102]. The circulating ANGPTL8 in humans and mice is predominantly liver-derived, with a minor proportion coming from adipose tissue [101]. Within the ANGPTLs family, ANGPTL8 has been shown to act as a hepatokine involved in hypothalamic appetite control [45]. ANGPTL8 levels were reduced by fasting [103] and increased upon refeeding [104]. Both peripheral and central ANGPTL8 administration reduce c-Fos positive neuronal expression in the dorsomedial hypothalamus (DMH), and alter neuropeptide-Y (NPY) activity in the hypothalamus, thus significantly reducing food intake [48]. ANGPTL8 is expressed in a range of appetite-related hypothalamic nuclei, including the paraventricular nucleus of the hypothalamus (PVN), DMH, the ventromedial hypothalamus (VMH), and the arcuate nucleus (ARC) [48] (Figure 2c). However, receptors for ANGPTL8 have not been definitively identified [45,105], and further research is warranted to clarify the mechanisms by which ANGPTL8 regulates metabolic activity across the liver–brain axis.

## 4. Metabolites from the Liver to the Brain

The role of the metabolites within the liver–brain axis can be summed up in several aspects: (1) Multiple hepatic metabolites, such as bile acids and ammonia, require secondary metabolism by the gut microbiota before entering the bloodstream and brain. (2) Gut-derived metabolites, such as short-chain fatty acids and gut hormones, act both in the liver and brain. (3) The metabolites of the liver itself, without intestinal secondary metabolism, affect the brain after intestinal absorption into the bloodstream, affecting nutrients such as bilirubin, choline, and vitamins.

### 4.1. Bile Acids

The portal vein, which collects blood from the gut, spleen, and pancreas, and the bile ducts, which contain bile secreted by the liver into the intestine, establish an anatomical bidirectional circulation along the liver–gut axis [106]. Bile acids (BAs) are the most well-known metabolites produced by the liver for secondary intestinal metabolism. Bile acids enter the systemic circulation, bind to plasma proteins, mainly albumin and lipoproteins [107], and distribute throughout the non-enterohepatic organs [108], such as the brain, heart, and muscles. Despite their low concentration in the brain, BAs play a key role in the regulation of central metabolic and immunological homeostasis [109]. Emerging evidence suggests that organs like the brain may participate in alternative BA synthetic pathways. For instance, CYP39A1, a cytochrome P450 in the brain, can convert cholesterol to oxysterols [110], which can be utilized for primary BA synthesis in the liver [111]. In the brain, the BA receptor, Takeda G protein-coupled receptor 5 (TGR5), is expressed in neurons, microglia, and astrocytes [112]. BAs, acting directly or indirectly on the brain via TGR5, have been shown to regulate food intake and mood. Physiological feeding in mice upregulates the concentration of BAs in the hypothalamus for a short time and specifically activates the expression of AgRP/NPY neuron membrane TGR5, thereby regulating the appetite [113,114]. Chronic stress has been linked to reduced TGR5 expression in the lateral hypothalamic area (LHA), and TGR5 agonists have been shown to modulate depression-like behavior through specific neural circuits [115]. Furthermore, TGR5 agonists exhibit anti-inflammatory and neuroprotective properties, and BAs are implicated in neurodegenerative diseases [116,117,118], hepatic encephalopathy [119] and amyotrophic lateral sclerosis (ALS) [120]. The role of BA metabolism in cognitive function and brain aging is an area of growing interest, with elevated serum conjugated primary bile acid (CPBAs) and ammonia observed in the elderly and individuals with cognitive impairment [121], while inhibiting intestinal bile acid absorption can alleviate cognitive decline in aged rodents [121].

### 4.2. Short-Chain Fatty Acids

A well-described effect of the gut microflora, with implications for CNS disease and therefore possibly affecting the liver–brain axis, is the production of short-chain fatty acids (SCFAs). SCFAs that are not taken up by colonic cells are transported into the portal vein. In the liver, all three SCFAs (butyrate, propionate, and acetate) serve as energy substrates for hepatocytes [122]. Only a small proportion of acetic, propionic, and butyric acids (36%, 9%, and 2%, respectively) from the colon reaches the circulatory system and parenteral tissues [123]. Sometimes the vagal afferent is a new route where metabolites execute the remote control of brain functions. SCFAs have been demonstrated to activate vagal afferent neurons, hence suppressing food intake [124].

SCFAs can cross the BBB to reach the brain, potentially facilitated by the monocarboxylate transporters (MCT) on endothelial cells [125]. Six SCFA receptors have been identified [126], and notably, GPR109A expression has been detected in the hypothalamic neuron [127] and rostroventrolateral medulla [128]. In several neurodegenerative illnesses, the concentrations of combinations of SCFAs and their corresponding gut flora are altered. SCFAs can modulate CNS immune responses by regulating microglia and T cells [129], regulate protein misfolding and accumulation, and improve cognitive impairment by rescuing mitochondrial dysfunction in the brains of diabetic mice [130], thereby affecting neurodegenerative diseases [126]. There are, however, two facets of the effects of SCFAs on the brain: the protective effects form the majority and the harmful effects form the minority [129]. Relevant experiments need to refine the composition and concentration of SCFAs that reach the brain.

### 4.3. Ammonia

About 90% of the total amount of ammonia produced by the intestine (about 4 g per day in adults) comes from bacterial urease-mediated urea hydrolysis [131]. Ammonia, continuously generated from amino acid breakdown in tissues, is efficiently converted to urea by the liver, with low blood concentrations [132]. When liver dysfunction occurs, ammonia cannot be metabolized into urea in the liver [133]. It enters the blood circulation from the intestine, resulting in increased blood ammonia [134]. Ammonia is neurotoxic, and the brain is one of the most vulnerable organs to the deleterious effects of ammonium [135,136]. Hepatic encephalopathy, characterized by an altered mental state and cognitive impairment, is a clinical manifestation of ammonia toxicity. Elevated ammonia levels in the blood and brain were observed in AD patients [137] and aged mice [121]. Previous studies have revealed that excessive ammonia exacerbates brain pathology. Interestingly, recent studies have found that ammonia can relieve stress and soothe mood, potentially through enhancing glutamine availability and supplementing presynaptic GABAergic neurotransmission [131].

### 4.4. Bilirubin

Roughly 80% of bilirubin is from the disintegration of senescent red blood cells by mononuclear phagocyte systems of liver, spleen, and bone marrow and from prematurely destroyed erythroid cells in the bone marrow. Unconjugated bilirubin travels with the blood stream to the liver, which converts unconjugated bilirubin into conjugated forms for bile secretion. Any obstacle in the process of bilirubin metabolism, such as the destroyed integrity of the BBB and the low plasma albumin, will cause bilirubin in the plasma to increase (hyperbilirubinemia) [138], commonly seen in neonates [139]. Free bilirubin passes through the BBB and is deposited in brain regions, inhibiting the utilization of oxygen by brain tissue and causing irreversible damage to the nervous system, a condition known as bilirubin encephalopathy [140].

### 4.5. Vitamin

The liver and bile salts play a crucial role in the absorption, storage, and metabolic transformation of fat-soluble vitamins A, D, E, K, and B12 [141,142]. When the liver is dysfunctional, vitamins synthesized by other organs cannot be absorbed, stored, and transformed by the liver, resulting in vitamin deficiency.

Vitamins are indispensable for brain development and function, with deficiencies implicated in degenerative diseases. Each vitamin is actively carried across the BBB [143]. B vitamins, in particular, are vital for neurotransmitter synthesis and brain physiological functions [144], with their concentration in the brain being 50 times higher than in the bloodstream [145]. Additionally, the turnover rate of B vitamins in the brain is considerable, from 8% to 100% every day [21]. Vitamin B6, as a coenzyme, deals in the biosynthesis of neurotransmitters including dopamine, serotonin, and GABA, and it exerts a neuroprotective influence on the glutamate system [146,147]. The principal role of vitamin B12 in neuropathy is attributed to myelin synthesis, which facilitates peripheral nerve regeneration [148,149]. Niacin, also known as vitamin B3, is an essential micronutrient for the synthesis of nicotinamide adenine dinucleotide (NAD) [150]. As a precursor of NAD+, niacin may be involved in the brain aging process.

The impact of vitamin C on the central nervous system, though less studied, is believed to be significant in curbing excessive inflammatory responses [151]. A deficiency in vitamin C can lead to the hyperactivity of the microglia, resulting in the release of numerous inflammatory mediators and the potential onset of neurological disorders and neurodegeneration.

The nexus between vitamin D and neurodegenerative diseases has been extensively studied in recent years. Population-based observational studies [152] and controlled trials [153] have suggested that vitamin D supplementation could offer benefits against dementia and AD. However, conflicting findings from animal and longitudinal studies have shown that vitamin D supplementation might exacerbate the progression of AD and increase mortality risk [154]. These divergent outcomes prompt us to pay attention to the dosage of vitamin D and course of vitamin D treatment on the CNS.

### 4.6. Choline

Choline is an essential nutrient found in various foods and serves as a precursor for the synthesis of betaine, choline phospholipids, and acetylcholine [155]. Choline absorbed by the brain may first enter a storage pool, possibly phosphatidylcholine in the membrane, and then be converted to acetylcholine. The human body can produce a modest amount of choline in the liver [156], yet the intake of this nutrient from external sources is imperative to avert deficiency symptoms. Current dietary recommendations for choline intake, 425 mg/day for adult women and 550 mg/day for adult men [156], may not be optimal for brain health, and inadequate intake has been correlated with an increased risk of dementia [157].

A large body of evidence underscores the importance of choline for maintaining healthy brain function [158,159]. Dietary choline deficiency developed AD symptoms and disrupted hippocampal networks in mice [159]. The vitality of choline in maintaining the brain health of humans starts prenatally and continues into maturity and old age. Randomized controlled experiments have demonstrated that increasing maternal choline consumption has long-lasting positive effects on children’s attention, memory, and problem-solving abilities throughout their school years [160]. In an AD mouse model, maternal supplementation with a 4.5-fold adequate daily intake of choline has shown improvements in spatial memory for offspring [161]. Additionally, choline supplementation in adult AD mice significantly reduced Aβ plaque density and brain inflammation [162,163].

### 4.7. Liver–Brain Axis and Gut Metabolites: Possible Association

**Figure 3:**
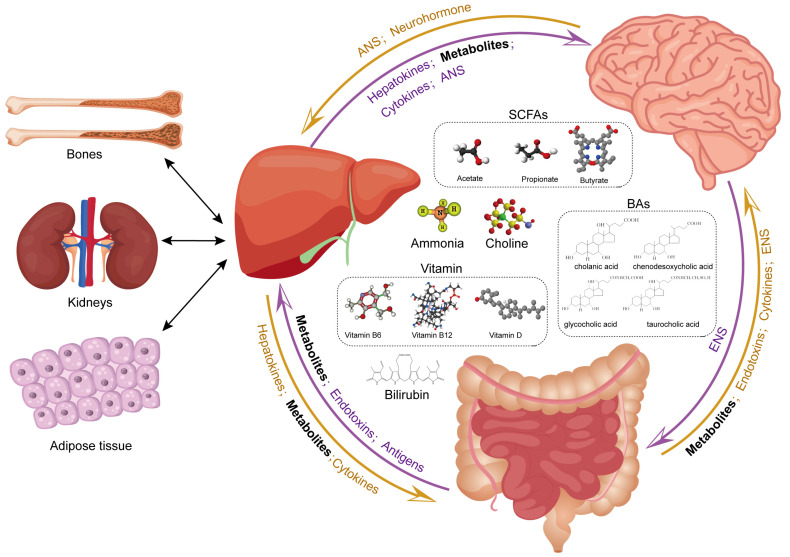
Distant communicating pathways, such as hormonal, neuronal, metabolic, and other factors, across liver-gut–brain axis. ANS, autonomic nervous system; ENS, enteric nervous system; SCFAs, short-chain fatty acids; BAs, bile acids. Part of this figure was drawn using materials from vecteezy.com, accessed on 20 May 2024.

The gut–liver–brain axis influences the development of disease, including changes in liver metabolites, microbial metabolites, intestinal permeability, endotoxins, antigens, cytokines, neurotransmitters, gut hormones, the intestinal enteric nervous system, and hepatic autonomic nerves. By taking into account the extensive research on the gut–brain axis [164,165], Figure 3 elucidated the role of liver-derived metabolites in the brain and explored mechanisms of bidirectional crosstalk between the liver–brain axis and the gut. 

The absorption and utilization of the food from the gut to the brain passes through the liver. The influence of gut microbiota metabolites on liver [166] and neurological [167,168] diseases is widely established. Metabolites produced by the liver are further processed by gut microflora and then absorbed into the blood through the intestine. Disruptions at any stage of the gut barrier—whether microbial, epithelial, or vascular—can challenge gut-liver crosstalk and trigger liver diseases such as NAFLD, alcoholic liver disease (ALD), and primary sclerosing cholangitis (PSC) [166]. As a result of which, the gut microflora has implications for liver and CNS disease, and, therefore, may also affect the liver–brain axis [169].

Enterohepatic circulation carries a variety of factors and metabolites to mediate communication between liver and intestine. Most metabolites of the intestine and liver can reach the brain via the blood flow; in addition to this, the autonomic nervous system of the intestine and liver sends information to the brain. The liver harbors a multitude of enzymes, some unique to it; for example, enzyme systems that synthesize ketone bodies and urea play a critical role in synthesizing essential metabolites that underpin the vitality of gut and brain. In addition to being produced in the brain, several neurotransmitters, such as serotonin, dopamine, and norepinephrine, are also produced by several bacteria present in the human gut microbiome. Although gut-produced neurotransmitter metabolites cannot directly cross the BBB, peripherally produced neurotransmitters may subsequently alter brain chemistry via vagus nerve stimulation. The hepatic vagus nerve works by sensing the intestinal microenvironment and providing sensory input to the brainstem nuclei [170].

The use of antibiotics, probiotics, and polyphenols confirmed the gut–liver–brain axis’ key role in different diseases. Some specific treatments, such as TGR5 agonists, FXR agonists, GLP-1 receptor antagonists, and FGF21 analogues, have beneficial effects on maintaining the balance of the gut–liver–brain axis [171]. Metabolomics has revealed a plethora of metabolites along the liver-gut–brain axis, yet few have a special effect on the brain. The further development of drug targets along the gut–liver–brain axis may be a key pathway for neuroprotection and metabolic improvement. Addressing this challenge necessitates a combination of liver secretomics, gut microbial metabolomics and cerebrospinal fluid metabolomics.

## 5. Neural Interfaces between Brain and Liver

Hepatic metabolism is regulated by humoral factors and neuronal activity. Even though humoral effects have long been considered predominant, the role of neuronal activity in liver metabolism is equally significant. The first report suggesting that liver metabolism could be affected by the CNS might be Claude Bernard’s experiment in 1849 that a puncture of the fourth ventricle caused temporary glycosuria [172]. Subsequent research has indicated that local neural networks are integral to liver pathologies such as NAFLD and hepatic insulin resistance [173]. Hypothalamic nuclei project to the brainstem and spinal cord, where they launch sympathetic or parasympathetic outflow to the liver. The liver also sends metabolic signals to the CNS via afferent nerves [174]. With the development of molecular neurobiology, neural pathways connecting the CNS to the liver are gradually elucidated. 

### 5.1. Hypothalamic Nuclei Influence Liver Metabolism

#### 5.1.1. Hypothalamic Nuclei and Liver

The hypothalamus can be artificially divided into several sections [175,176]. Stanley et al. applied a combination of viral and transgenic techniques to locate and describe neural populations that project from the hypothalamus to the liver [177], including the following: arcuate nucleus (ARC), suprachiasmatic nucleus, paraventricular nucleus, lateral hypothalamus (LH), dorsomedial hypothalamus (DMH) of hypothalamus, dorsal motor nucleus of vagus, pontine reticular nucleus, nucleus of the solitary tract, nucleus ambiguus, paraventricular thalamus, and the central amygdaloid nucleus. These hypothalamic nuclei, which interface with the liver, are labeled with abbreviations in Table 2. Neurons that compose these nuclei are sensitive to either signals from the peripheral nervous system or circulating stimuli, such as fluctuations in nutrients and hormone levels. They collectively establish an appetite set point, a baseline for food intake, to modulate hepatic glucolipid metabolism [178].

#### 5.1.2. ARC and Liver Metabolism

There are two groups of best-studied intermingled neurons in the ARC, the agouti-related peptide (AgRP) neurons and the pro-opiomelanocortin (POMC) neurons, both of which control fat accumulation and hepatic glucose production in opposite ways. POMC is a precursor protein of the anorexigenic α-melanocyte-stimulating hormone (α-MSH), which reduces food intake by activating the MC4R expressed by target neurons [179]. AgRP neurons release two orexigenic neuropeptides, AgRP and neuropeptide (NPY) [180], generally promoting feeding. Hormones, including insulin, ghrelin, leptin, and cholecystokinin, alter the activity of these neurons to affect glucose metabolism [181,182]. Subsets of POMC and AgRP neurons are also excited or inhibited by plasma glucose levels [183]. POMC activation improves hepatic insulin sensitivity. Conversely, AgRP activation decreases hepatic insulin sensitivity.

#### 5.1.3. PVN and Liver Metabolism

PVN resides in the center of the hypothalamus and integrates a variety of signals from different brain regions, including ARC, VMH, SCN, and LH [184]. Then, the preganglionic neurons receive information from the PVN to adjust the metabolic activity of the autonomic pathway [184]. Gao et al. discovered that in animal models of type 2 diabetes, there is a general shift towards excitation in hypothalamic nuclei associated with the liver [185]. Among these shifts, alterations in autonomic circuits in PVN are key factors in the imbalance of the brain–liver autonomic nerve pathway, which contributes to dysregulated liver functions [185].

#### 5.1.4. VMH and Liver Metabolism

The VMH was initially identified as a key hypothalamic site for energy homeostasis. Steroidogenic factor-1 (SF-1) neurons, a population of VMH glutamatergic neurons, are vital for peripheral metabolic homeostasis [186]. The optogenetic stimulation of SF-1 neurons raises hepatic glucose production [187] and simultaneously enhances hepatic insulin sensitivity. The calcium channel subunit and thrombospondin receptor alpha2delta-1 (α2δ-1) regulate the activity of SF-1 neurons through non-canonical mechanisms [188]. SF-1 neurons exert concomitant effects on sympathetic output by projecting to the anterior bed nucleus of the stria terminalis, thus affecting blood glucose levels [189].

### 5.2. Nerve Fiber Connections between the CNS and the Liver

**Figure 4:**
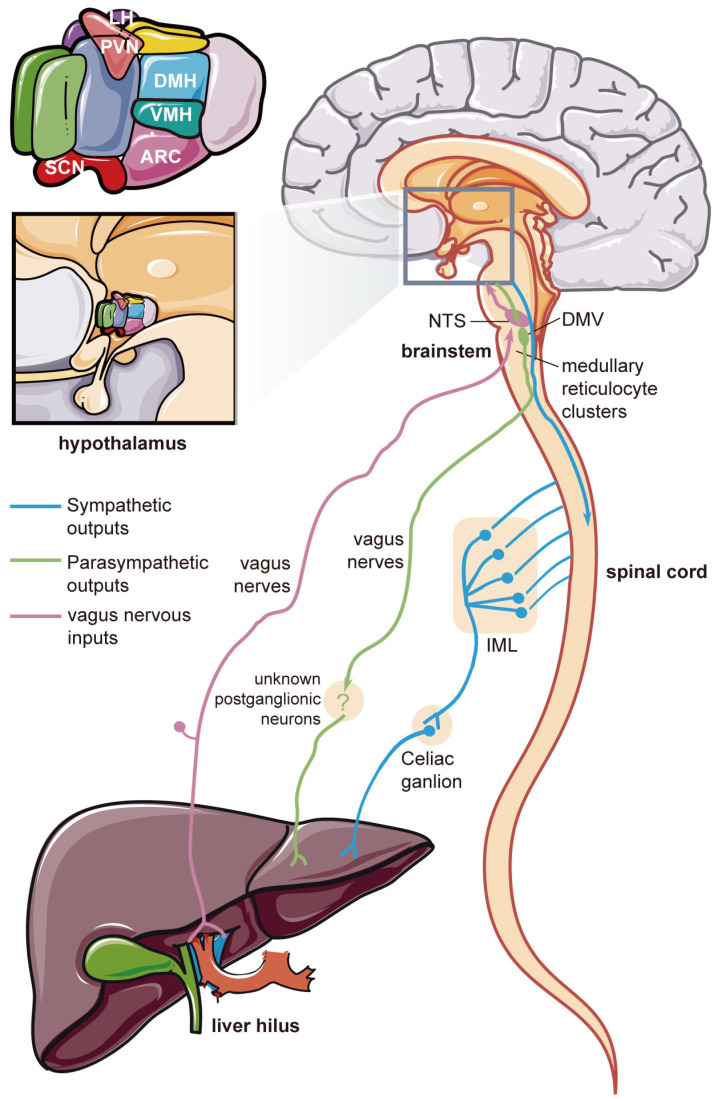
Nerve fiber connections between the brain and the liver. NTS, nucleus of the solitary tract; DMV, dorsal motor nucleus of the vagus; IML, intermediolateral column. Part of this figure was drawn using materials from smart.servier.com, accessed on 3 February 2024.

The hypothalamic control of feeding behavior and liver metabolism relies on the autonomic nervous system (ANS) (Figure 4). The route of nerve impulses between the brain and the liver is categorized into efferent and afferent nerves.

#### 5.2.1. Afferent Sensory Nerves

Macronutrients absorbed from gastrointestinal digestion enter the liver through the portal vein. For a long time, the liver’s metabolic sensing was deemed the primary source of metabolic signals to the brain, since the hepatoportal system contains a large number of chemoreceptors. The sensory receptors in the portal vein and liver transmit hepatic metabolic feedback to the brain via the vagus nerve [190]. Feeding increases the level of glucose in both the portal vein and intrahepatic blood; concurrently, the excitement of the vagus nerve fosters higher hepatic glucose absorption and facilitates glycogen synthesis. When the hepatic branch of the vagus nerve is severed, glucose and glycogen metabolism regulation gets impaired [191]. Currently, the liver’s metabolic sensing role is being reevaluated as hormone receptors in the brain, such as insulin [192], leptin and ghrelin, which allow the brain to sense blood-borne metabolic information from peripheral organs.

#### 5.2.2. Efferent Nerve Pathways

The efferent nerve pathways from the brain to liver are composed of two branches of the ANS, namely the sympathetic and parasympathetic systems. The divisions of parasympathetic and sympathetic nerves are the yin and yang of the ANS [193]. For hepatic metabolism, the activation of sympathetic aminergic and peptidergic innervation in the liver results in gluconeogenesis. Meanwhile, parasympathetic activity increases carbohydrate storage and lowers hepatic glucose output [194], even though the presence of parasympathetic nerve endings in the liver is challenged.

For the sympathetic hepatic nerve, the pre-autonomic neurons in the hypothalamus emit hypothalamomedullary fibers [195] that ultimately terminate in the intermediolateral column (IML) of the spinal cord. These fibers go through the periaqueductal gray and adjacent reticular formation of the brainstem before reaching the IML [196]. In addition, the hypothalamus also sends direct projects to the sympathetic preganglionic neurons of the IML [197] via the hypothalamospinal fibers [198]. Preganglionic neurons originating from IML in the lateral horn of the thoracolumbar spinal cord extend their axons into the celiac ganglion (postganglionic neurons), which innervate the liver. Postsynaptic sympathetic hepatic nerve bundles penetrate into the liver to varying degrees, accompanied by portal hepatic vessels [196].

The efferent parasympathetic autonomic signals are transmitted through preganglionic cells in the dorsal motor nucleus of the vagus (DMV) of the brainstem and nearby medullary reticulocyte clusters. DMV is directly connected to postganglionic ganglion cells via the vagus nerve, without involving the spinal cord. The postganglion cells are presumably located in the vicinity of the liver. How intrahepatic nerve fibers connect to exogenous nerves remains unclear so far [199], and the presence of parasympathetic intrahepatic fibers is debatable. Recently, a team demonstrated the presence of only symmetric nerves and no parasympathetic neurons in different mammalian liver tissue substances through systematic observations of the three-dimensional distribution of nerves in mice, monkeys, and human liver [200]. Subsequently, sympathetic neurodegenerative lesions were found in the liver in different mouse obesity models. For this reason, it is sympathetic rather than parasympathetic nerves in the liver that affect the process of metabolic disorders.

#### 5.2.3. Neurohormone and the Liver

Although the liver is not the primary site of action for most neurohormones, it plays a significant role in the inactivation of neurohormones. This part of the evidence is slightly old, and for the completeness of the review, it will be briefly described here. Neurohormones known to be effectively inactivated in the liver include neurohypophyseal antidiuretic hormone (ADH), prolactin (PRL) [201], growth hormone (GH), gonadotropins (Gn), and melanocyte-stimulating hormone (MSH). In instances of experimental liver damage or human liver pathology, water retention is frequently reported, which is often attributed to impaired ADH inactivation by the liver [202].

## 6. Advanced Techniques for Tracing and Transmitting along the Liver–Brain Axis

**Figure 5:**
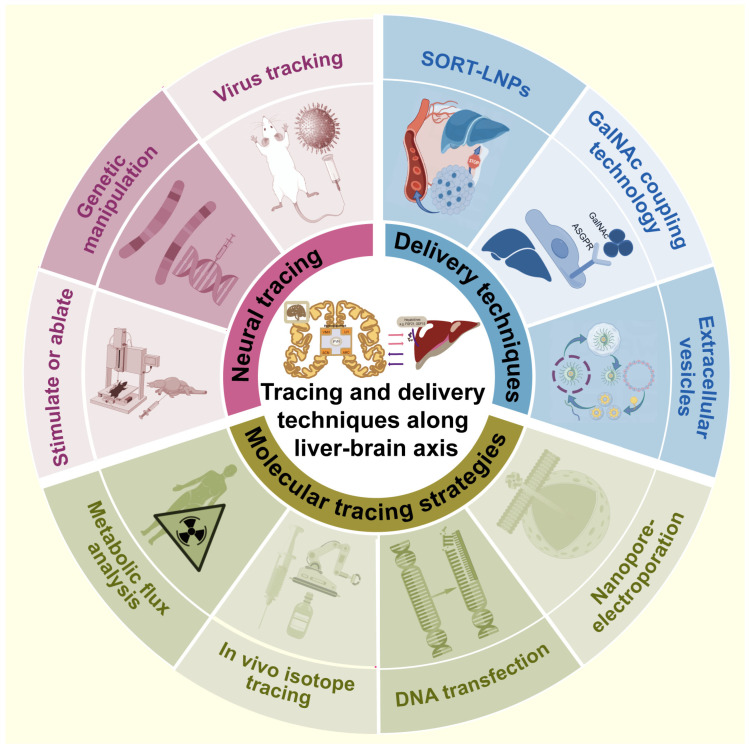
Strategies to observe crosstalk between liver and brain, including tracing strategies to neural circuits and hormonal molecules, and some molecular delivery techniques from liver to brain. SORT-LNPs, selective organ targeting-lipid nanoparticles; GalNAc, N-acetylgalactosamine coupling; ASGPR, Asialoglycoprotein receptor. Part of this figure was drawn using materials from https://www.figdraw.com/#/, accessed on 20 May 2024.

The delineation of the molecular mechanisms of metabolic crosstalk along the liver–brain axis remains in its early stages. The development of tracing and delivery techniques is essential for accelerating discoveries in this area. This part will cover molecular tracing in systemic circulation, viruses for retrograde neuronal circuits, and organ-specific drug carriers targeting the liver or brain (Figure 5).

### 6.1. Molecular Tracing

Most of the humoral factors from liver to brain are small-molecule metabolites and proteins. The metabolomics of the liver and the brain reflect their static metabolite abundance. Metabolic flux analysis (MFA) can also reveal the turnover flux of metabolites in the circulatory system [203] and the origin of intermediate metabolites in different tissues [204], facilitating the identification of interorgan metabolic exchange. In vivo isotope tracing combined with metabolic flow analysis offers dynamic insight into metabolic processes [205,206,207].

Eukaryotic metabolism is characterized by tissue and cell heterogeneity, but single-cell and single-organelle metabolomics have not yet been established. After oral, intravenous, or intraperitoneal injection of isotope tracers, liver tissue, portal blood, brain tissue, and cerebrospinal fluid were obtained. To compensate for the low resolution of current metabolomics techniques, prior to metabolic quenching and metabolite extraction, fluorescence-activated cell sorting or mass spectrometry imaging spatial metabolomics can be used to characterize metabolite levels and isotope labeling patterns at subcellular resolution. The current method, based on mass spectrometry imaging (MSI), needs a necessary trade-off in spatial resolution, metabolite coverage, and sensitivity. However, these methods only represent the average value of metabolic intermediate labeling within the tissue [203]. Neither isotope tracing nor MFA can track individual molecules.

Single-molecule tracking allows for studying the ins and outs of metabolic molecules in the liver–brain axis. A certain degree of single-molecule tracking can be achieved by photoactivatable or photoconvertible fluorescent fusion proteins, inorganic fluorescence probes, and membrane-permeable dyes, which are integrated with in vivo imaging technology [208,209]. Although DNA transfection is already widely used, the direct delivery of proteins into cells will be more effective at tracking single molecular metabolic pathways [210]. The nanopore electroporation technique holds great potential in the field of intracellular single-molecule imaging to deliver proteins labeled with organic dyes into living cells. For example, in in vitro experiments, hepato-intestinal metabolites can be delivered into neurons using nano-electroporation technology; in these in vivo experiments, the target hepatogenic protein is labeled by tail vein injection, and the expression of the label is observed by radiographic techniques or in brain sections.

### 6.2. Neural Tracing

Before the advent of modern neuroanatomy, scientists studied neural projections between the CNS and visceral tissues by stimulating specific brain areas or ablating autonomic nerves. This expands our knowledge of how the central site controls liver metabolism. Limitations include the inability to visualize neural connections and the risk of damaging the surrounding regions [211].

Actually, viruses that infect neurons across synapses, particularly the rabies virus, have been the most common method for retrograde neuronal circuits [212]. It is the advent of virus tracking that has promoted the study of viscera-specific projections originating from the brain. In rodent models for functional validation, genetic manipulation techniques for specific neuronal populations help to delineate the central sites innervating peripheral organs [213]. The combination of virus tracking and transgenic strategies may facilitate studies of metabolic coordination of organs that appear anatomically disparate but functionally related [177]. Injecting retrograde viruses into the liver allows us to explore the neural connections between the liver and the brain. The role of target molecules in the liver and brain can be explored through the strategy of liver- or brain-specific knockout of target genes. However, due to their low throughput, genetic manipulation techniques are mainly employed for confirming connections proposed by other methods.

### 6.3. Transmitting Techniques

With the rapid development of drug delivery technologies, organ-specific carriers are just around the corner. SORT-LNPs are reportedly tissue-specific mRNA delivery platforms that introduce selective organ targeting (SORT) nanoparticles into conventional lipid nanoparticles (LNPs), which breaks the liver accumulation limit for drug delivery to extrahepatic tissues [214,215]. On the other hand, N-acetylgalactosamine (GalNAc) coupling technology has significant advantages in treating liver diseases. GalNAc has been identified as a targeted ligand with a high affinity for the Asialoglycoprotein receptor (ASGPR) [216], specifically highly expressed on hepatocyte surfaces. In contrast, the receptor is expressed much less on other cell surfaces [217]. Therefore, this technology can exclusively focus on metabolic disorders in the liver and rarely enter other tissues.

In the past few years, extracellular vesicles (EVs), especially exosomes, have attracted considerable attention as novel delivery vehicles for drugs [218]. Once released in circulation, exosomes can reach any organ where they transmit signals to their recipient cells with or without direct cell-to-cell contact [219]. Despite exosome-regulated metabolic signaling across the liver–pancreas [220], liver–fat [221] and fat–brain [222] axes, no studies have detailed the mechanisms by which exosomes regulate metabolism via the hepatic–brain axis. EVs are often described as biomarkers and novel delivery systems for therapeutic agents in metabolic liver diseases [223,224] and neurodegenerative diseases [225]. As natural nanoparticles in systematic circulation, exosomes may be ideal drug delivery vectors from the liver to brain due to their lower immunogenicity, their longer circulation time in body fluids, and their ability to cross the BBB compared to synthetic carriers.

## 7. Summary and Perspectives

The regulation of physiological activities and the pathology of metabolism-related diseases in the brain and liver depend on the metabolic signals across the liver–brain axis. This intricate communication is mediated by the systemic circulation and ANS, which together orchestrate the metabolic crosstalk between the liver and the brain. Herein, we encapsulated the current knowledge of metabolic signals across the liver–brain axis from the perspective of interconnected diseases of liver and brain, hepatokines, metabolites, nervous connections, and neurohormones. Unraveling the molecular mechanisms linking humoral factors and neural pathways across the liver–brain axis is of substantial importance for advancing our understanding of metabolic-related diseases, contributing to a holistic view of disease pathology.

Hepatokines and metabolites have emerged as promising targets for drug discovery aimed at treating liver and brain pathologies. Notably, FGF21 and GDF15 are advancing into clinical trials, holding potential for the treatment of obesity and its associated comorbidities. Despite these advances, research into the liver–brain axis remains nascent, with many questions yet to be resolved. Future research needs to address the following questions:

(1) While certain liver-derived factors are known to be recruited to the CNS, only a few of these circulating factors have been identified for their central receptors and central action. It is worth continuing to study the effects of liver factors on the central nervous system and the sites of action, and trying to find specific drugs that regulate the central metabolism.
(2) Intestinal flora metabolites mediate liver–brain interactions. The complexity of the gut microbiome is daunting. Most of the current research is an observational snapshot of the gut microbiota and has not explored in detail the dynamic evolution of microbial products in the liver and brain. How to determine the source of intestinal metabolites? What role does the liver play in these processes? What is the dynamic evolution of gut microbes in different tissues? These questions are left for future studies.
(3) Though the central location of receptors for several liver factors has been identified, delivering drugs to specific brain regions and avoiding side effects are still a challenge. Future studies could explore discovering more hepatogenic molecules with central receptor and regulatory roles to develop highly selective agonists or antagonists for the neuroregulation of metabolism.
(4) The hypothalamus–ANS–liver axis has been confirmed, with several hypothalamic nuclei engaging in hepatic metabolism through ANS outputs. However, the precise neuroanatomy and the transmission of metabolic information via nerve fibers in the liver require further refinement.
(5) Cutting-edge techniques such as single-molecule tracking and cell type-specific transgenic methods will be instrumental in deciphering how the liver communicates with the brain. However, the throughput and resolution are low, and we call for the development of single-cell and single-organelle metabolomics technologies.

## Figures and Tables

**Table 2:** The subdivisions of the hypothalamus and the functions of the nuclei associated with liver metabolism.

From Medial to Lateral	From Anterior to Posterior	Hypothalamic Nuclei	Abbreviation	Functions on Liver Metabolism
Periventricular zone		Periventricular nucleus		
		Suprachiasmatic nucleus	SCN	SCN manipulates the circadian clock of hepatic glucose secretion.
		Arcuate nucleus	ARC	ARC receives integrated information from the VMH and LH about food intake. AgRP neurons and POMC neurons control fat accumulation and hepatic glucose production in opposite ways.
Intermediate zone	Preoptic area	Periventricular nucleus		
		Medial preoptic nucleus		
		Lateral preoptic nucleus		
	Supraoptic area (anterior area)	Suprachiasmatic nucleus	SCN	/
		Supraoptic nucleus		
		Paraventricular nucleus	PVN	PVN integrates multiple signals from different brain areas including ARC, VMH, SCN and LH.
		Anterior hypothalamic nucleus		
		Lateral hypothalamic nucleus	LH	LH serves as a “feeding center” and is involved in modulating feeding behavior.
	Tuberal area (middle area)	Arcuate nucleus	ARC	/
		Dorsomedial nucleus	DMH	DMH integrates feeding behavior with circadian activity.
		Ventromedial nucleus	VMH	VMH is involved in feeding behavior and is said to be a “satiety center”.
		Lateral tuberal nucleus		
	Mammillary area (posterior area)	Mammillary nucleus		
		Posterior hypothalamic nucleus		
		Lateral hypothalamic nucleus	LH	/
Latera zone		Lateral preoptic nucleus		
		Lateral tuberal nucleus		
		Lateral hypothalamic nucleus	LH	/

## Data Availability

No new data were created or analyzed in this study. Data sharing is not applicable to this article.

## Abbreviations

AD: Alzheimer’s disease; AgRP: agouti-related peptide; AHCD: acquired hepatocerebral degeneration; ANGPTL: angiopoietin-like proteins; ANS: autonomic nervous system; AP: area postrema; APOE: apolipoprotein E; APP: acute-phase proteins; ARC: arcuate nucleus; Aβ: amyloid-β; BAs: bile acids; CNS: central nervous system; DMH: dorsal medial nucleus; DMV: dorsal motor nucleus of the vagus; EVs: extracellular vesicles; FGF: fibroblast growth factor; GDF: growth differentiation factor; GFRAL: glial-derived neurotropic factor receptor-alike; GHSR: growth hormone secretagogue receptor; HE: hepatic encephalopathy; HNF: hepatocyte nuclear factor; HPA: hypothalamic–pituitary–adrenal; IGF-1: insulin-like growth factor 1; IGF1R: insulin-like growth factor 1 receptor; IML: intermediolateral column; LCN2: lipocalin-2; LEAP2: liver-enriched antimicrobial peptide-2; LH: lateral nucleus; LILRB3: leukocyte immunoglobulin-like receptor B3; LPL: lipoprotein lipase; LRP-1: low-density lipoprotein receptor-related protein 1; LXR: liver X receptor; MC4R: melanocortin-4 receptor; MFA: metabolic flow analysis; NAFLD: non-alcoholic fatty liver disease; NAG: nonsteroidal anti-inflammatory drug-activated gene; NASH: non-alcoholic steatohepatitis; NPY: neuropeptide Y; NTS: nucleus of the solitary tract; POMC: pro-opiomelanocortin; PPARα: peroxisome proliferator-activated receptor alpha; PVN: paraventricular nucleus; RET: rearranged during transfection; SCFAs: short-chain fatty acids; SCN: suprachiasmatic nucleus; sEH: soluble epoxide hydrolase; SF-1: steroidogenic factor-1; SLC22A17: solute carrier family 22 member 17; SREBP: sterol-regulatory element binding proteins; TBI: traumatic brain injury; TGR5: Takeda G protein-coupled receptor 5; TSK: Tsukushi; VMH: ventral medial nucleus.
